# Early vascular ageing phenotypes and urinary targeted metabolomics in children and young adults: the ExAMIN Youth SA and African-PREDICT studies

**DOI:** 10.1007/s00726-023-03293-2

**Published:** 2023-06-17

**Authors:** Ashleigh Craig, Ruan Kruger, Lebo F. Gafane-Matemane, Roan Louw, Carina M. C. Mels

**Affiliations:** 1 Hypertension in Africa Research Team (HART), North-West University, Private Bag X6001, Potchefstroom, 2520 South Africa; 2 MRC Research Unit for Hypertension and Cardiovascular Disease, North-West University, Potchefstroom, South Africa; 3 Human Metabolomics, North-West University, Potchefstroom Campus, Potchefstroom, South Africa

**Keywords:** Adults, Arterial stiffness, Children, Early vascular ageing, Targeted metabolomics

## Abstract

**S**ome individuals are susceptible to accelerated biological ageing, resulting in premature alterations in arterial structure and function. Identifying early-onset vascular ageing characterised by arterial stiffening is vital for intervention and preventive strategies. We stratified and phenotyped healthy children (5–9 yrs) and young adults (20–30 yrs) into their vascular ageing extremes established by carotid-femoral pulse wave velocity (cfPWV) percentiles (i.e., healthy vascular ageing (HVA) and early vascular ageing (EVA)). We compared anthropometric, cardiovascular, and metabolomic profiles and explored associations between cfPWV and urinary metabolites. Children and adults in the EVA groups displayed higher levels of adiposity, cardiovascular, and lifestyle risk factors (adults only) (all *p* ≤ 0.018). In adults, several urinary metabolites were lower in the EVA group (all *q ≤ *0.039) when compared to the HVA group, with no differences observed in children. In multiple regression analysis (adults only), we found inverse associations between cfPWV with histidine (adj. *R*^2^ = 0.038; *β = *–0.192; *p* = 0.013) and beta-alanine (adj. R^2^ = 0.034; *β = *–0.181; *p* = 0.019) in the EVA group, but with arginine (adj. R^2^ = 0.021; *β = *–0.160; *p* = 0.024) in the HVA group. The inverse associations of beta-alanine and histidine with cfPWV in the EVA group is suggestive that asymptomatic young adults who present with an altered metabolomic and less desired cardiovascular profile in combination with unfavourable lifestyle behaviours may be predisposed to early-onset vascular ageing. Taken together, screening on both a phenotypic and metabolic level may prove important in the early detection, prevention, and intervention of advanced biological ageing.

**Supplementary Information:**

The online version contains supplementary material available at 10.1007/s00726-023-03293-2.

## Introduction

Even in apparently healthy individuals, arterial changes occur throughout the cardiovascular (CV) system where such changes (thickening and stiffening of large arteries) (Lakatta et al. 2003; Nagai et al. 1998) may be present in the absence of hypertension (Pearson et al. 1997). Measures of arterial stiffness, such as the gold standard carotid-femoral pulse wave velocity (cfPWV) increase across the lifespan (Liang et al. 2019). In this context, arterial stiffness is thought to reflect cumulative damage due to CV risk factors (Willum-Hansen et al. 2006). Together with the irreversible ageing process, these arterial changes represent an intermediate step in the development of cardiovascular disease (CVD) later in life (Willum-Hansen et al. 2006). In some young individuals, biological ageing seems to take a more rapid course resulting in premature alterations in arterial structure and function (Nilsson et al. 2009; Olsen et al. 2016). Such observations led to the concept of early vascular ageing (EVA) (Nilsson et al. 2009).

Early vascular ageing is present in individuals who experience arterial stiffening as seen by heightened cfPWV (≥ 85th cfPWV percentile) or with multiple risk factors (i.e., hypertension (O’Rourke et al. 1999), obesity (Wildman et al. 2003), smoking, and alcohol consumption (Charakida et al. 2019)) associated with arterial stiffness (Bruno et al. 2020). Conceivably, identifying markers related to EVA, ideally prior to the onset of CVD, may potentially aid in the detection of premature arterial damage and further prompt intervention and preventive strategies to address potentiating risk factors. This may, in part, delay a trajectory towards EVA in young individuals who are at risk. The mechanisms underlying the development of EVA, even in hypertensive individuals, remain unclear and may involve risk factors that influence normal metabolism and include a combination of CV, socioeconomic, sociocultural, and/or epigenetic factors (Nilsson 2015).

Metabolomics offers a powerful platform to investigate metabolic changes that are associated with several pathophysiological states (Newgard 2017). Significant correlations have been reported between several metabolites (i.e., 4-hydroxyproline, alanine, glutamine, glycine, histidine, and serine) and central systolic blood pressure (cSBP) in black South African adults (Mels et al. 2019), known to be at higher risk for EVA (Schutte et al. 2020; Kruger et al. 2021). Another study in black South African boys found an inverse association between beta-alanine and PWV, suggesting this metabolite may be partly involved in the early onset of arterial stiffness in this population (Erasmus et al. 2018). Indeed, there have been several more studies proving that metabolomics provides important insight into CVD (De Beer et al. 2020; Nikolic et al. 2014; Tzoulaki et al. 2018).

Our study was, therefore, in part, motivated by the fact that metabolomic studies in healthy children and young adults are limited and warrants further exploration. To the best of our knowledge, no single cross-sectional study has focussed on early metabolomic changes related to arterial stiffness indices in a study comprising of both children and young adults. Previous work from our research team highlighted associations between BP, left ventricular mass index and several non-essential amino acids and recommended future studies should investigate the relation between other measures of CV structure and function (Mels et al. 2019; Erasmus et al. 2018). Thus, the latter formed the second part of the motivation for this current study. Healthy South African children (5–9yrs) and young adults (20–30yrs) were stratified into low cfPWV (healthy vascular ageing (HVA), i.e., ≤ 15th cfPWV percentile) and high cfPWV (EVA risk, i.e., ≥ 85th cfPWV percentile), and metabolomics profiles compared within each age cohort. We further explored associations between cfPWV and urinary metabolites in children and young adults.

## Methodology

**Fig. 1:**
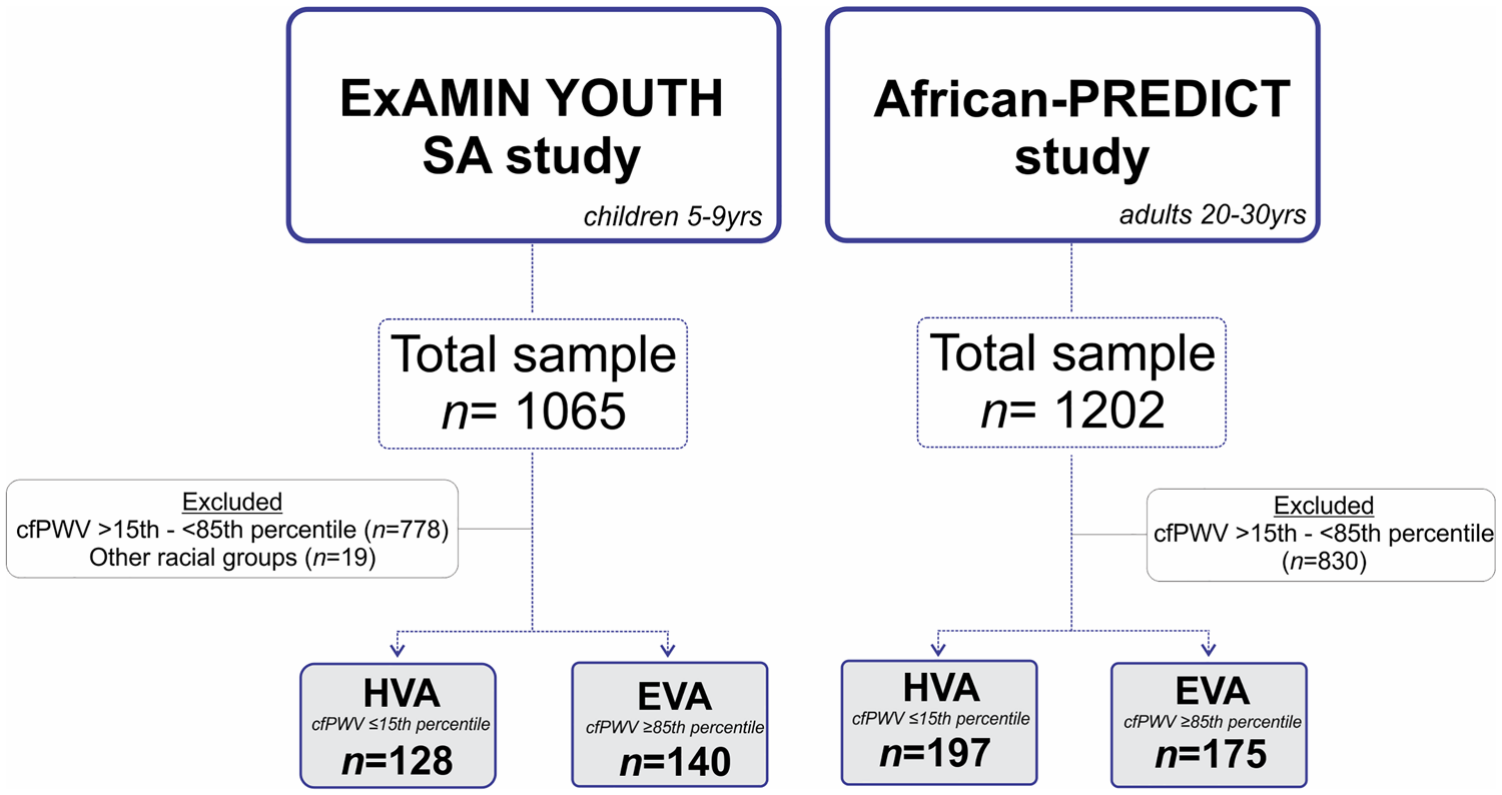
Sample size and stratification of both study populations used in this cross-sectional study

We included data of apparently healthy children (aged 5–9yrs) from the Exercise, Arterial Modulation and Nutrition in Youth South Africa (ExAMIN Youth SA) study and young adults (aged 20–30yrs) from the African Prospective study on Early Detection and Identification of Cardiovascular disease and Hypertension (African-PREDICT) study who had complete CV and metabolomic datasets. We further stratified both study populations according to different vascular ageing profiles, i.e., HVA, and those at risk of EVA as outlined in Fig. 1 (ExAMIN Youth SA: *n* = 268; African-PREDICT: *n* = 372) (Bruno et al. 2020).

The study population and protocol for both the ExAMIN Youth SA study (Kruger et al. 2020) and the African-PREDICT study (Schutte et al. 2019) have been described elsewhere. Briefly, the ExAMIN Youth SA study included children of both sexes and all races residing within the Dr. Kenneth Kaunda district who voluntarily (with parental permission) wanted to participate. This study was designed to investigate the relationship between body composition, dietary intake, physical fitness, and physical activity, psychosocial stress, CV function as well as urinary and salivary biomarkers. There were no specific exclusion criteria; however, children were excluded if no informed consent from the parent was obtained or if the child did not feel comfortable participating. For the current analysis, only black and white children were included due to a lack of statistical power in the group of children from other race groups (Indian and coloured children; *n* = 19).

Exclusion criteria for the African-PREDICT study, included office blood pressure (BP) > 140/90 mmHg, or with any self-reported diseases or risk factors that may influence CV health, internal ear temperature > 37.5 °C, human immunodeficiency virus, diabetes mellitus, liver disease, cancer, tuberculosis, or renal disease as well as the use of chronic medication. Pregnant and lactating women were also excluded due to known influences of hormones on CV health (O’Kelly et al. 2022).

Both studies were conducted in line with the ethical principles of the Declaration of Helsinki (Carlson et al. 2004) and were approved by the Health Research Ethics Committee of the North-West University. Additionally, both the African-PREDICT (NCT03292094) and the ExAMIN Youth SA (NCT04056377) studies are registered at ClinicalTrials.gov. All participants, including their parents (for the children participants), were fully informed about the objectives of the study, and written informed consent/assent was acquired from each participant.

### Questionnaires

In the African-PREDICT study, basic demographic information was collected with the use of a General Health and Demographic Questionnaire. This questionnaire is seen as a self-administered screening tool that was completed prior to participation. The following information was gathered from the questionnaire: age, sex, ethnicity, and current lifestyle risk factors such as self-reported smoking and alcohol consumption. In the statistical analyses, age (years) was used as a continuous variable, while sex (0 = women; 1 = male), ethnicity (0 = black; 1 = white) and lifestyle risk factors (0 = no; 1 = yes) were captured as binary variables.

### Anthropometric measures

All anthropometric procedures, including weight and height were performed according to specific guidelines set out by the International Society for the Advancement of Kinanthropometry (ISAK) (Schutte et al. 2019; Stewart A et al. 2011). Body mass index (BMI) (weight (kg)/square height (m^2^)) of each participant was then calculated (SECA portable 213 stadiometer; SECA 813 electronic scale; Birmingham, UK). Body mass index z-scores were used for the assessment of body composition in children. Thresholds derived from a child growth reference was used to classify the BMI z-scores of children according to their age and sex (Cole et al. 2005).

### Cardiovascular measures

In the ExAMIN Youth SA study, brachial BP was measured in triplicate on the upper right arm at heart level with a validated automated oscillometric pediatric BP monitor (Omron HBP-1100-E; OMRON HealthCare Co., LTD. Kyoto, Japan) (El Assaad et al. 2003). Measurements were conducted five times with 1-min intervals on the right arm. The three measurements with the smallest variation were used to calculate a mean. With the use of a Dinamap^®^ ProCare 100 Vital Signs Monitor, brachial BP of the African-PREDICT participants was measured on the left arm, thereafter on the right arm in duplicate followed by a repeated measure on the left arm (GE Medical Systems, Milwaukee, USA). In this study, the left BP measurement was used in the analyses. Systolic blood pressure (SBP) and diastolic blood pressure (DBP) were captured from each measurement. The mean arterial pressure (MAP) was subsequently calculated from brachial BP recordings. Mean arterial pressure was calculated using the formula: (DBP)/(0.4*pulse pressure) (Kiers et al. 2008).

Pulse wave analysis in the ExAMIN Youth SA study was performed using the validated oscillometric Mobilo-O-Graph monitor (I.E.M GmbH, Germany) and integrated with ARCSolver software (Wassertheurer et al. 2010; Weiss et al. 2012). The appropriately sized pediatric BP cuff was placed on the right upper arm of the children participant, from which the cfPWV was measured while children remained in a seated position. The African-PREDICT study made use of the SphygmoCor XCEL device (AtCor Medical Pty. Ltd., Sydney, New South Wales, Australia) to preform pulse wave analysis in adult participants (Van Bortel et al. 2012). Adults were required to remain in supine position, the right carotid artery was located by means of palpation to identify the strongest pulse point. The carotid pulse was measured using a tonometer while the femoral pulse was measured by a femoral cuff placed around the thigh of the participant. The transit-distance method was used and 80% of the distance calculated and entered after which the cfPWV was measured along the descending thoracic abdominal aorta using the foot-to-foot velocity method. In both the ExAMIN Youth SA study and the African-PREDICT study, duplicate measurements were taken and the mean value of the two closet measures was used in subsequent analyses. Any measurements not considered of sufficient quality were repeated based on an operator index and additional quality indices reflecting the degree of variation above acceptable limits (Townsend et al. 2015).

Since cfPWV is a recognised marker of EVA (Bruno et al. 2020), we used the measurement to stratify our sample population according to different vascular ageing profiles, i.e., HVA (≤ 15th cfPWV percentile), and those at risk of EVA (≥ 85th cfPWV percentile).

### Biochemical analyses

The ExAMIN Youth SA children were required to provide a first void mid-stream urine sample in the privacy of their own home with assistance from their parents. Children participants were provided with sealable urine containers to collect first urine samples on the day of participation. Children participants were required to bring their urine sample on the day of participation. Participants of the African-PREDICT study were required to provide an early morning spot urine sample at the Hypertension Research and Training Clinic of the North-West University. All samples were prepared, aliquoted into cryo-vials and stored in bio-freezers (–80 °C) until analysed.

### Metabolomic data collection

Metabolomics data were collected using a liquid chromatography–tandem mass spectrometry (LC–MS/MS) method using an Agilent© system (1200 series LC front end coupled to a 6410 series triple quadrupole mass analyser) with electrospray ionisation source operated in positive ionisation mode (Chen et al. 2013). The methods used to analyse the metabolomic data used in this current study has been described elsewhere (Du Toit et al. 2022). Briefly, urine samples were randomised and analysed in batches of 20 samples per batch. Three quality control urine samples and an additional in-house standard mixture (consisting of all analysed metabolites) were analysed with each batch. The in-house standard mixture was used to ensure data integrity. Urine samples of a predetermined volume (corresponding to 0.25 μmoles creatinine, to compensate for variation in urine concentrations) were defrosted overnight at 4 °C after which an isotope mixture comprising of several amino acid (lysine, valine, isoleucine, phenylalanine) and acylcarnities (acetylcarnitine, octanoylcarnitine, octadecanoylcarnitine) isotopes (100 μL; 2.5 ppm; dissolved in acetonitrile), serving as internal standards, were added. Thereafter, the sample was vortex mixed (15 s) and kept at − 20 °C for 20 min. The samples were further centrifuged at 15,000 × g for 10 min and the supernatant transferred to an Agilent glass vial. The samples were then dried under nitrogen (37 °C) and stored (-80 °C) until analysis. Before analysis the samples were defrosted and dried under nitrogen (37 °C, 25 min). After the addition of 300 μL butanolic hydrochloric acid [4:1 (v/v) n-butanol:acetyl chloride], samples were incubated at 50 °C for 1 h and dried again under nitrogen (37 °C). The dried residue was reconstituted in a final volume (100 μL) of water:acetonitrile (50:50, v/v) containing 0.1% formic acid. The samples were then centrifuged followed by the transfer of the supernatant (70 μL) to an Agilent glass vial containing a 250 μL pulled point glass insert. For the separation of metabolites, a Zorbax SB-Aq 80 Å StableBond column (Agilent^©^, 2.1 mm × 100 mm × 1.8 μ; cat# 828,700–914) with Zorbax Eclipse Plus C18 guard column (Agilent^©^, 2.1 mm x 5 mm, 1.8 μm, cat# 821,725–901) were used. The column was kept at 45 °C during the entire run, with an injection volume of 0.3 μL. The chromatographic gradient started at 95% solvent A (water with 0.1% formic acid) with a flow of 0.3 mL/minute maintained for 0.2 min before the gradient were increased to 25% solvent B (acetonitrile with 0.1% formic acid) at 2 min. The gradient was kept constant for 5 min, after which it was increased linearly to 90% solvent B at 7.5 min. These conditions were maintained for 1.6 min, with the flow linearly increased to 0.4 mL/min between 9 to 9.1 min. Thereafter, the gradient was increased to 95% solvent B at 12 min and kept constant for 1 min, followed by decreasing the gradient to 5% solvent B at 13 min. A post run of 5 min ensured equilibration of the column. Mass spectrometry was operated in multiple reaction monitoring mode. Electrospray ionisation source gas (nitrogen) temperature was kept at 320 °C, with a flow rate of 10 L/minute. Nebuliser pressure were kept at 30 psi and the capillary voltage at 3500 V. Regarding data prepossessing, a peak intensity filter was applied to remove features with areas below the limit of quantification (LOQ cut-off of area < 750). Metabolomics data were then normalised to the added isotope internal standards. Furthermore, spectral data matrices were individually inspected for each batch to ensure good data quality. This included: manual data inspection; visual inspection of multivariate data clustering (including quality control samples) via principal component analysis; univariate inspection of metabolite intra- and inter-batch coefficient of variance distributions, as well as scatter plots of relative intensity vs. run order for both individual samples and metabolites. Altogether, the data proved to be good quality with no batch effects visible. Urinary metabolites are reported as arbitrary units (AU).

### Statistical analyses

For statistical analyses, IBM^®^ SPSS^®^ version 27 (IBM Corporation, Armonk, New York) and GraphPad Prism version 5.03 for Microsoft^®^ Windows (GraphPad Software, San Diego, California, USA) were used to analyse and plot the data. Variables were tested for normality using the Kolmogorov–Smirnov test and QQ-plots. Metabolomic variables were logarithmically transformed. Data was expressed as mean ± standard deviation if normally distributed and as geometric mean with 5^th^ and 95^th^ percentile boundaries for skewed variables.

For comparisons between the groups, independent *T* tests (adjustment for multiple comparisons were carried out to lower the false discovery rate for all metabolites (*q* < 0.05)) and analysis of covariance (ANCOVA) were used with adjustments applied for MAP (when cfPWV was a dependent variable). Pearson and partial (adjusted for age, sex, ethnicity, and MAP) correlations were used to determine the relationships of cfPWV and urinary metabolites. Standard multiple regression analyses were conducted with cfPWV as a dependent variable and tested for associations with urinary metabolites (adjustments made for age, sex, ethnicity, BMI and MAP). Additionally, standard multiple regression analyses were conducted to confirm associations between cfPWV and urinary metabolites, with adjustments made for age, sex, ethnicity, BMI, self-reported smoking, alcohol consumption and MAP.

## Results

The general characteristics of both study populations stratified by their vascular ageing profiles are presented in Table 1. Children in the EVA risk group were slightly (by 6 months) older (*p* < 0.001), and predominately of white ethnicity (*p* < 0.001). Adults in the EVA group were older (*p* < 0.001) (by 18 months), predominately male (*p* < 0.001) and of black ethnicity (*p* = 0.003). In young adults, those stratified in the EVA risk group displayed similar characteristics to the children in the EVA risk group, i.e., body composition (height, waist circumference and BMI), and BP (SBP, DBP and MAP) all higher in those in the EVA risk groups when compared to those in the HVA groups (all *p* ≤ 0.018). In young adults (5.17 m/s vs 7.75 m/s) and children (4.00 m/s vs 4.95 m/s), cfPWV was also significantly different between the HVA and EVA groups (p < 0.001). Furthermore, in adults, self-reported smoking and alcohol consumption were higher in the EVA risk group (both *p* < 0.001).

**Table 1:** General characteristics of the children and adult study population stratified according to cfPWV percentiles

	African-PREDICT			ExAMIN Youth SA		
	Lower 15th percentile (cfPWV ≥ 5.5 m/s) (*n* = 197)	Upper 85th percentile (cfPWV ≤ 7.2 m/s) (*n* = 175)	*p* value	Lower 15th percentile (cfPWV ≥ 4.10 m/s) (*n* = 128)	Upper 85th percentile (cfPWV ≤ 4.80 m/s) (*n* = 140)	*p* value
Age (years)	23.9 ± 3.13	25.4 ± 2.98	** < 0.001**	7.12 ± 0.996	7.78 ± 0.796	**< 0.001**
Sex (male *n* (%))	29 (14.7%)	147 (84.0%)	** < 0.001**	57 (44.5%)	68 (45.6%)	0.30
Ethnicity (black *n* (%))	89 (45.2%)	105 (60.0%)	**0.003**	100 (78.1%)	77 (55.0%)	** < 0.001**
Anthropometry						
Body height (cm)	164 ± 8.03	174 ± 9.05	** < 0.001**	119 ± 774	127 ± 7.55	** < 0.001**
Body weight (kg)	69.9 ± 16.7	72.7 ± 17.2	**0.14**	21.8 ± 4.48	28.6 ± 7.53	** < 0.001**
Waist circumference (cm)	78.7 ± 11.7	81.7 ± 11.9	**0.018**	51.9 ± 5.70	58.9 ± 8.77	** < 0.001**
Body mass index (kg/m^2^)	26.0 ± 5.72	24.0 ± 4.67	**0.001**	15.3 ± 1.93	17.6 ± 3.19	** < 0.001**
Body mass index z-score	–	–	–	–0.414 (–2.16; 1.29)	0.376 (–1.57; 2.16)	** < 0.001**
Blood pressure measures				0.417	3.08	
Brachial SBP (mmHg)	111 ± 10	127 ± 12	** < 0.001**	94 ± 8	111 ± 10	** < 0.001**
Brachial DBP (mmHg)	73 ± 7	85 ± 8	** < 0.001**	60 ± 6	69 ± 8	** < 0.001**
Mean arterial pressure (mmHg)	88 ± 8	102 ± 9	** < 0.001**	74 ± 6	86 ± 8	** < 0.001**
Cardiovascular measures						
Pulse wave velocity (m/s)^†^	5.17 ± 0.34	7.75 ± 0.94	** < 0.001**	4.00 ± 0.135	4.95 ± 0.157	** < 0.001**
Self-reported						
Smoking (yes/no)	28 (14.2%)	63 (36.0%)	** < 0.001**	–	–	–
Alcohol use (yes/no)	87 (44.2%)	110 (62.9%)	** < 0.001**	–	–	–

**Fig. 2:**
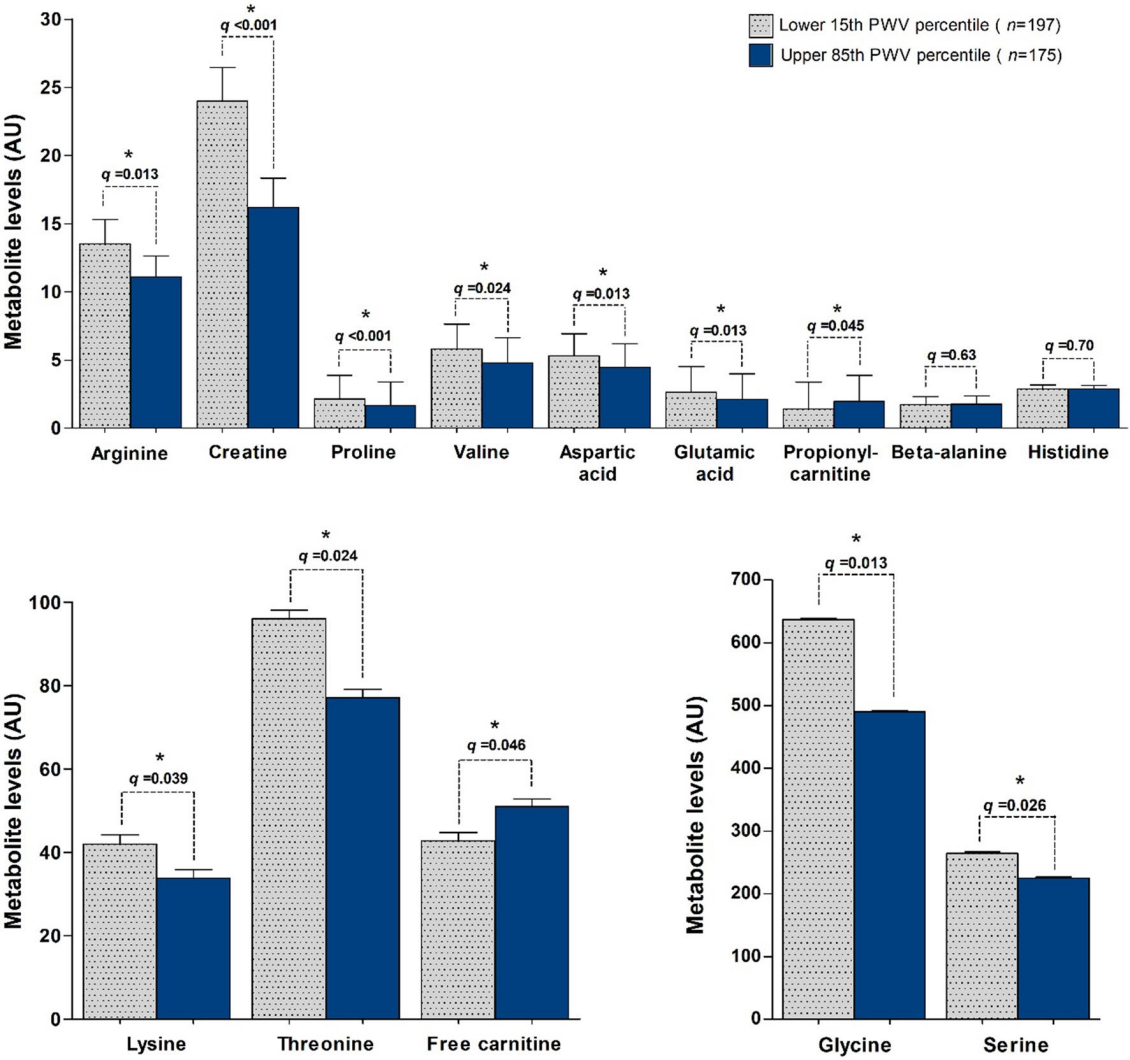
A comparison of identified metabolomics (AU) (mean and SD) in adults (African PREDICT study) between the lower 15th cfPWV percentile and the upper 85th cfPWV percentile. Adjustment for multiple comparisons were carried out to lower the false discovery rate for all metabolites. *Indicates statistical significance after adjustment of multiple comparisons (*q ≤ *0.05)

Several urinary metabolites were compared across the vascular ageing groups between both children and young adults (Supplementary Table S1) while adjusting for multiple comparisons (Fig. 2). In young adults, arginine, creatine, proline, valine, aspartic acid, glutamic acid, lysine, threonine, glycine, and serine (*q ≤ *0.039) were lower in the EVA risk group when compared to the HVA group. Additionally in young adults, when compared to the HVA group, free carnitine and propionylcarnitine (*q ≤ *0.046) were higher in the EVA risk group. No significant differences in metabolomic profiles were observed in children.

**Fig. 3:**
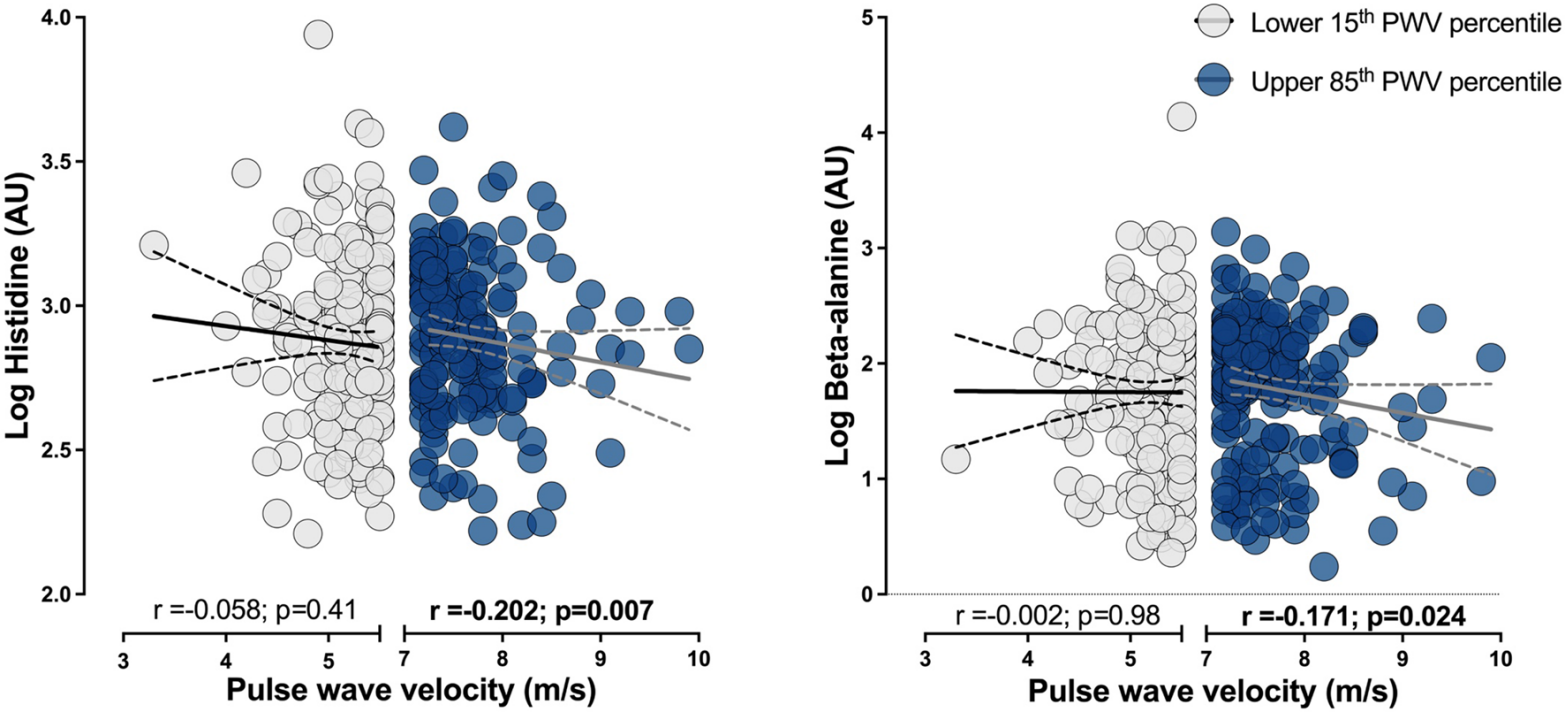
A scatterplot illustrating the bivariate correlation of cfPWV with histidine and beta-alanine in young adults (African-PREDICT) stratified by cfPWV percentiles (lower PWV ( HVA, i.e., ≤ 15th cfPWV percentile) and high PWV (EVA risk, i.e., ≥ 85th cfPWV percentile))

In bivariate (Fig. 3 and Supplementary Table S2) and partial regression (Supplementary Table S3) analyses (adjusted for age, sex, ethnicity, and MAP), in young adults, both histidine (*r = *–0.199, *p* = 0.009) and beta-alanine (*r = *–0.183, *p* = 0.016) associated inversely with cfPWV in the EVA risk group only. Arginine was the only metabolite found in young adults to associate with cfPWV in the HVA group (*r = *–0.170, *p* = 0.018), but only after adjustments for age, sex, ethnicity, and MAP were applied.

**Fig. 4:**
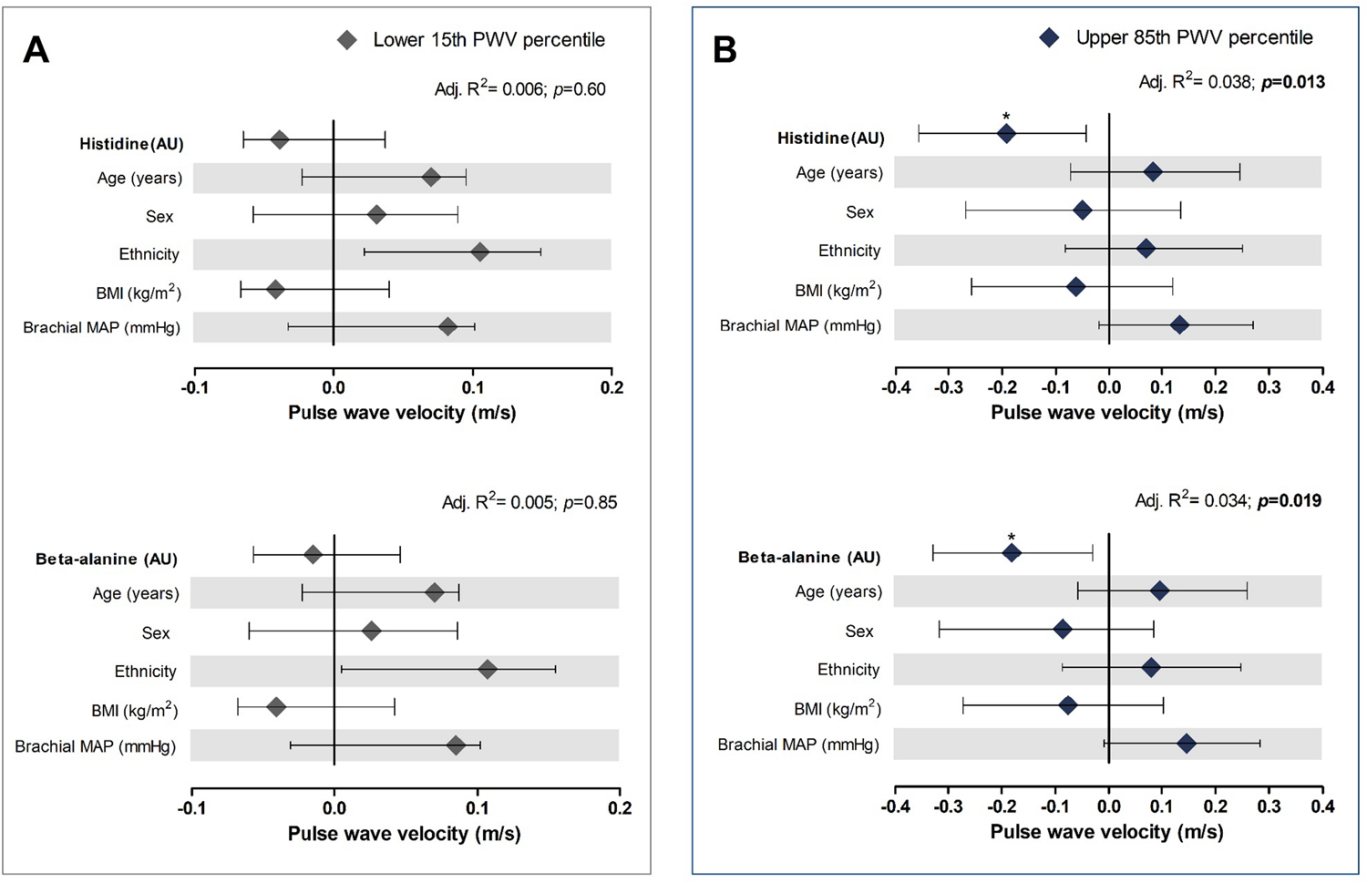
Multiple regression anaylses of carotid-femoral pulse wave velocity with respective metabolites in A) lower 15^th^ cfPWV percentile and B) upper 85^th^ cfPWV percentile

In standard multiple regression analysis, we found consistent inverse associations (Fig. 4, Supplementary Table S4 and Table 2) of cfPWV with histidine (adj. R^2^ = 0.038; *β = *–0.192; *p* = 0.013) and beta-alanine (adj. *R*^2^ = 0.034; *β = *–0.181; *p* = 0.019) in young adults in the EVA risk group. Additionally, in young adults, cfPWV associated inversely with arginine in the HVA (adj. *R*^2^ = 0.021; *β = *–0.160; *p* = 0.024) group. No significance was reached after multiple adjustments in the children.

**Table 2:** Unadjusted standard multiple regression analyses with metabolites and cfPWV in adults (African-PREDICT) stratified by cfPWV percentiles

	Pulse wave velocity (m/s)					
	Lower 15th cfPWV Percentile (*n* = 197)			Upper 85th cfPWV Percentile (*n* = 175)		
	Adjusted R^2^	Std β (95% Cl)	*p* value	Adjusted R^2^	Std β (95% Cl)	*p* value
Histidine (AU)	0.002	–0.058 (–0.071; 0.029)	0.41	0.035	–0.202 (–0.363; –0.057)	**0.007**
Beta-alanine (AU)	0.005	–0.002 (–0.050; 0.048)	0.98	0.024	–0.171 (–0.315; –0.023)	**0.024**
Arginine (AU)	0.021	–0.160 (–0.089; –0.006)	**0.024**	0.006	–0.106 (–0.293; 0.050)	0.16

*n* number of participants. Bold values denote statistical significance (*p* < 0.05).

We additionally performed a standard multiple regression analyses to investigate whether the reported associations of cfPWV with histidine and beta-alanine are influenced by lifestyle risk factors (Table 3), including self-reported smoking and alcohol consumption. The significant associations previously reported in adults in the EVA risk group (cfPWV and histidine: adj. *R*^2^ = 0.052; *β = *– 0.197; *p* = 0.011; cfPWV and beta-alanine: adj. *R*^2^ = 0.047; *β = *– 0.187; *p* = 0.019) remained robust.

**Table 3:** Adjusted standard multiple regression analyses with metabolites and pulse wave velocity in adults (African-PREDICT) stratified by cfPWV percentiles with adjustments made for priori covariates

	Pulse wave velocity (m/s)					
	Lower 15th cfPWV Percentile (*n* = 197)			Upper 85th cfPWV Percentile (*n* = 175)		
	Adjusted R^2^	Std β (95% Cl)	*p* value	Adjusted R^2^	Std β (95% Cl)	*p* value
Histidine (AU)	0.004	–0.036 (–0.036; 0.039)	0.63	0.052	–0.197 (–0.363; –0.047)	**0.011**
Beta-alanine (AU)	0.005	–0.015 (–0.057; 0.047)	0.84	0.047	–0.187 (–0.328; –0.030)	**0.019**

Variables included in the model were: age, sex, ethnicity, BMI, MAP, smoking and alcohol consumption. *n* number of participants. Bold values denote statistical significance (*p* < 0.05).

## Discussion

In this cross-sectional study, we found that children and adults in the EVA risk group presented with a less desired CV profile with higher BP and cfPWV measures, while adults were further found to have higher lifestyle risk factors such as smoking and alcohol consumption, when compared to those in the HVA group. Several metabolites (arginine, creatine, proline, valine, aspartic acid, glutamic acid, lysine, threonine, glycine, and serine) known to impact vascular function either through vasodilation (Craig et al. 2020), increasing vascularity (Durante 2020), protein synthesis (Gwin et al. 2020), muscle growth and repair (Gwin et al. 2020) and/or improving cardiac remodelling (Wang et al. 2016) were found to be lower in the adult EVA risk group when compared to the HVA group. Additionally, we found both histidine and beta-alanine to inversely associate with cfPWV in the adult EVA risk group only. These associations remained significant after adjustment for several important confounders known to be associated with vascular health, including demographics (age, sex and ethnicity), adiposity (BMI), and lifestyle risk factors (smoking and alcohol consumption), suggesting alterations in normal metabolism are associated with EVA. For adults, it is clear that the spread in cfPWV is larger when compared to the children. And perhaps the lower variance in cfPWV in the children may explain why we also did not see anything in the metabolomics data.

**Fig. 5:**
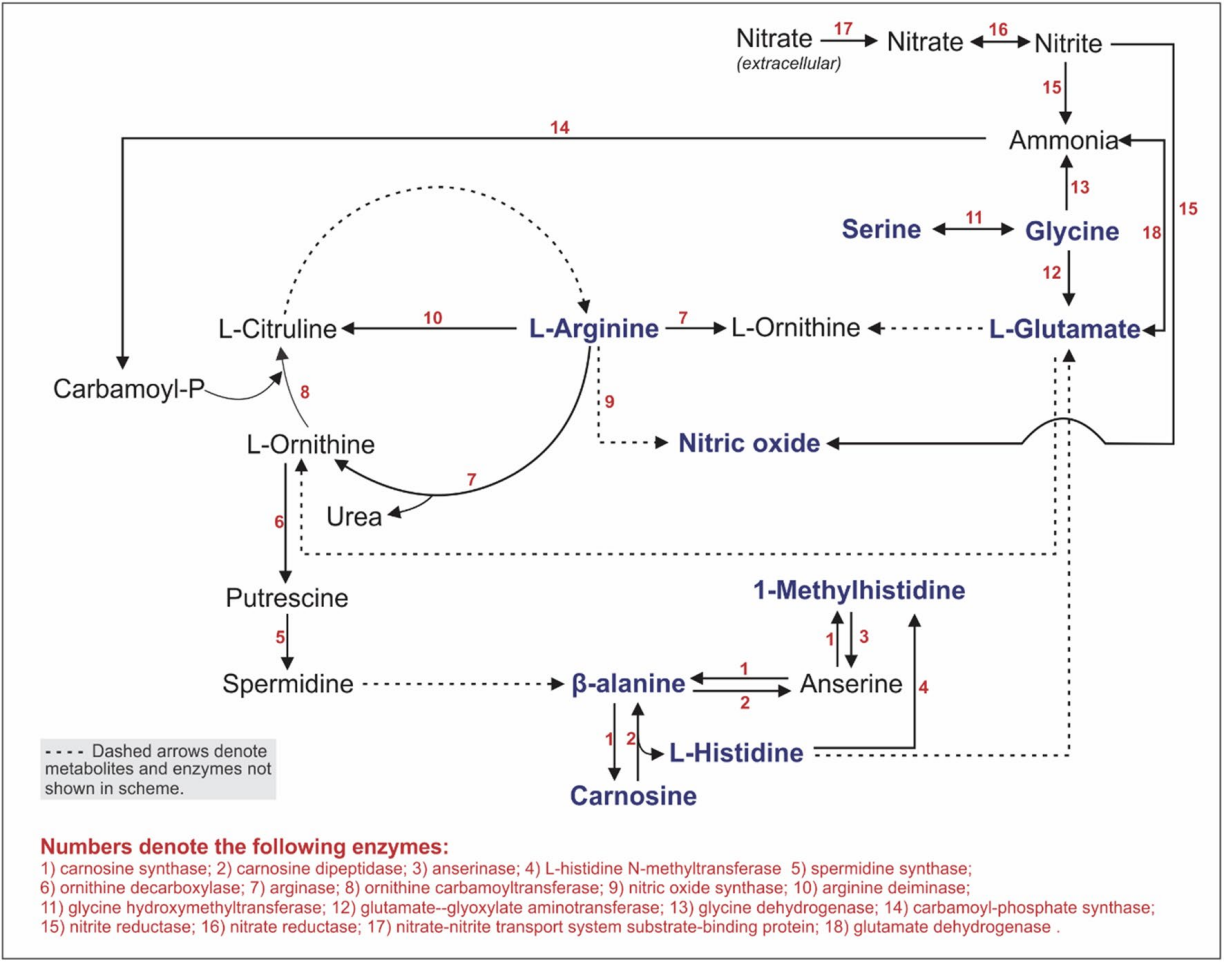
Illustration of the metabolic pathways involved in the main findings of this study. The urinary metabolite pathway scheme was derived from KEGG pathways showing the link between several essential and non-essential amino acids in this study (arginine, glycine, serine, glutamate, beta-alanine, histidine, carnosine) and nitric oxide, suggesting the plausible influence these amino acids have on nitric oxide bioavailability (i.e., homeostatic regulation of nitric oxide may be altered if the identified amino acids are found in low quantities thus resulting in vascular dysfunction)

On a metabolic level, in young apparently healthy adults, we found several metabolites to be lower in those participants in the EVA risk group, when compared to those in the HVA group, potentially contributing to the increase in BP and cfPWV, we observed in this group. Lower concentrations of both non-essential (arginine, proline, aspartic acid, glutamic acid, glycine, and serine) and essential (valine, lysine, and threonine) amino acids were found in this group, which is comparable to numerous studies in diseased populations such as those with hypertension (Wang et al. 2015), atherosclerosis (Tuel et al. 2009) and stroke (Jung et al. 2011). Arginine, glutamic acid (glutamate), and glycine modulate nitric oxide (NO) concentrations (Fig. 5), a potent vasodilator (Prasad et al. 1999; El Hafidi et al. 2006; Vasdev et al. 2009; Toba et al. 2010). Decreased synthesis and bioavailability of NO in early life has been implicated in the potential increased risk for future large artery stiffness and hypertension development in later life (Craig et al. 2020, 2021). In addition, former studies portrayed arginine, glutamic acid, and glycine as cardioprotective amino acids (Jennings et al. 2015) and dietary intake of these amino acids can lower BP (Dong et al. 2011; Stamler et al. 2009; Cziraki et al. 2020). The mechanisms by which these amino acids reduce BP is by either acting as a vasodilator aiding in the production of NO (arginine) (Cziraki et al. 2020); by strengthening the antioxidant capacity (glutamic acid/glutamate) (Zhao et al. 2019) and/or by participating in the reduction of free radical formation thus increasing NO bioavailability (glycine) (El Hafidi et al. 2006) (Fig. 5). Serine is also known to have BP lowering effects through aiding in vasodilation (Mishra et al. 2008). Both glycine, a primary amino acid in collagen–and serine are required for collagen biosynthesis (Nigdelioglu et al. 2016), and the cardioprotective effects of collagen in reducing arterial stiffness, increasing NO, and lessening markers related to vascular damage have been well described (Kouguchi et al. 2013). Branched chain amino acids such as valine are important biomarkers that have been shown to significantly associate with carotid intima media thickness, BMI, waist circumference, and BP, risk factors for coronary artery disease and diabetes (Yang et al. 2014; Wang et al. 2011). Furthermore, sufficient levels of another essential amino acid, lysine is known to prevent atherosclerotic plaque build-up in arteries, by obstructing lipoprotein attachment in the arterial walls, therefore reducing BP (Pauling 1991). Taken together, lower levels of both the non-essential and essential amino acids in this group could have potentially resulted in those presenting with increased BP and CV measures, ultimately resulting in those stratified in the EVA risk group most susceptible to early onset vascular ageing.

The most prominent finding of our study is the inverse associations we found between measures of arterial stiffness and both a non-essential (beta-alanine) and essential (histidine) amino acid in the adult EVA risk group, even though both these amino acids were found comparable between the groups. Studies have shown beta-alanine and 1-methylhistidine, a derivative of histidine, to be cardioprotective via the synthesis of the dipeptide carnosine (Wu et al. 2003; McCarty et al. 2014; Ivanov et al. 2007). Carnosine is known for its’ versatile antioxidant activity by serving efficiently as an electron donor, preventing lipid peroxidation (Kohen et al. 1988; Pavlov et al. 1993) and in skeletal and cardiac muscle, amplifies the impact of cytoplasmic calcium on muscular contraction (Dutka and Lamb 2004). The inverse association we report between cfPWV and beta-alanine in the EVA risk group is in line with a previous study which included children from the Arterial Stiffness in Offspring Study (ASOS) (Erasmus et al. 2018). Although this study did not look at the potential risk for the development of EVA, it confirmed the inverse association between PWV and beta-alanine in black boys (Erasmus et al. 2018)–a population well known for early onset arterial stiffness (Mokwatsi et al. 2017). In brief, it was, therefore, suggested that the benefits of beta-alanine are due to carnosine, its derivative (McCarty et al. 2014). The inverse association between cfPWV and beta-alanine are, therefore, suggestive of a reduced bioavailability of carnosine which, may in part, be involved in early onset arterial stiffness (Erasmus et al. 2018). Carnosine has also been shown to improve the lipid profile and inhibit the development of atherosclerosis (brown et al. 2014). The previous study by Erasmus et al. (2018), as in ours, observed that beta-alanine levels did not differ between the stratified groups (Erasmus et al. 2018). Taken together, it is suggestive that even when beta-alanine levels are comparable, a lessened cardioprotective effect of its derivative, carnosine may pre-exist (Erasmus et al. 2018).

Carotid-femoral PWV was also found to inversely associate with histidine in the adult EVA risk group. Our findings on the association of PWV with histidine is in accordance with a prior study that found an inverse association with central SBP and histidine in black men (Mels et al. 2019), considering the link between arterial stiffness and central BP has previously been established (Kohara et al. 2009). However, due to differences in study designs, these results may not be directly comparable. Histidine can be metabolised to form glutamate (Fig. 5), that binds to an important N-methyl-D-aspartate receptor (NMDAR) (Holecek 2020). Stimulation of NMDAR causes calcium ions and protein kinase C-mediated activation of NO synthase (NOS) leading to the formation of NO (Gunasekar et al. 1995). As discussed above, NO is a vital component in normal vascular function (Prasad et al. 1999) and this association consequently suggests that the inverse association between a marker of arterial stiffness and histidine may indicate that a decrease in histidine may consequently lead to a decreased formation of NO (Fig. 5), with consequent increased vasoconstriction (Tsai et al. 2006). The link between NO and arterial stiffness has previously been established (Craig et al. 2020, 2021). Taking into consideration the numerous CV functions that these amino acids exert (Mels et al. 2019), our results, therefore, suggest that a reduced bioavailability thereof, may have adverse effects on the vascular system (i.e., endothelial dysfunction (Clapp et al. 2004)) of those stratified in the EVA risk group, therefore, increasing arterial stiffness among those most susceptible.

We also report an inverse association between cfPWV and arginine in the HVA group. Another potential target to attenuate vascular ageing is the arginine metabolic pathway (Fig. 5) (Prasad et al. 1999). As arginine is a substrate for NOS, arginine not only has vital NO-dependent effects (vasodilatory and antithrombotic) but also several NO-independent effects that aid in the maintenance of CV health (Cziraki et al. 2020). Due to the latter, arginine has become a popular dietary supplement (Dong et al. 2011), although the CV benefits from long-term supplementation remain unknown. Arterial stiffness is regulated by the endothelium through the release of NO and, therefore, as expected, a decrease in the availability of particular substrates (i.e., arginine), is one of the proposed mechanisms implicated in the pathophysiology of altered endothelial function (Gokce et al. 2004). Seeing that central pressure is also known to reflect central arterial stiffness (Litwin et al. 2019), the inverse association we report between cfPWV and arginine is comparable to a previous study from our research unit that reported inverse associations between central BP and another NO substrate and homologue of arginine, namely plasma homoarginine (Craig et al. 2021). The inverse association between cfPWV and arginine, therefore, suggests a beneficial effect arginine exerts on vascular function, through increased NO bioavailability. Again, we speculate that dietary intake may play a role in arginine concentrations, however, further investigation is warranted.

Lastly, a comparison between the vascular ageing profiles, found that, in children and adults alike, body composition (waist circumference by ≥ 4%), BP (BP by ≥ 12%) and CV (cfPWV by ≥ 19%) measures were higher in the EVA risk group when compared to the HVA group. Accordingly, we expected those participants in the EVA risk group to have higher BP and CV measures, than those in the HVA group, as elevated BP exhibits arterial changes (Kotsis et al. 2011). Adults stratified in the EVA risk group also consistently showed a higher percentage of participants who smoke (by ≥ 21%) and consume alcohol (by ≥ 18%). This, therefore, suggests, based on a phenotypic profile, participants stratified in the EVA risk group, who are potentially susceptible to early onset vascular ageing, are those who generally have higher levels of adiposity, BP, and frequent unfavourable lifestyle behaviours.

To conclude, several essential and non-essential amino acids known to aid in vascular function were lower in those in the EVA risk group when compared to the HVA group. In the EVA risk group, we found both beta-alanine and histidine to inversely associate with arterial stiffness, while in the HVA group, arginine inversely associated with arterial stiffness. Our results, therefore, suggest that young apparently healthy individuals may be predisposed to the development of EVA, and this may potentially be extrapolated among those with an altered metabolomic profile, potential heightened CV risk and resultant NO dysregulation.

### Strengths, limitations and recommendations

This explorative study must be interpreted within the context of its strengths and limitations. This study was well planned and executed under strict conditions. Populations included participants from the North West Province of South Africa and are not representative of the population as a whole. This study is limited by its cross-sectional design; hence, we were unable to investigate precise mechanisms and causal relationships. This study also lacked the use of flow-mediated dilation to assess endothelial function and was limited as carnosine was not a metabolite measured. Moreover, due to the hypothesis-generating nature of this study, our findings of specific metabolites associating with arterial stiffness indices relating to NO should be investigated in future studies. Such studies should also consider dietary behaviour which may influence BP. The study was conducted under highly controlled conditions in a well-equipped research facility; however, future studies are warranted to explore specific metabolites through highly targeted assays to verify our hypothesis that certain metabolites can be seen as potentiating factors to identify high-risk individuals.

### Supplementary Information

Below is the link to the electronic supplementary material.

Supplementary file1 (DOCX 42 KB)

## Data Availability

All data supporting the findings of this study are available within the paper and its Supplementary Information.
