# Supplementary material for: Early vascular ageing phenotypes and urinary targeted metabolomics in children and young adults: the ExAMIN Youth SA and African-PREDICT studies
Source: Amino Acids. 2023 Jun 17;55(8):1049–62. doi: 10.1007/s00726-023-03293-2 (PMC10514129; doi:10.1007/s00726-023-03293-2)
Supplement: Supplementary file 1 — Supplementary file1 (DOCX 42 KB) [file 726_2023_3293_MOESM1_ESM.docx]

**Supplementary Table S1: Metabolomics data of the adult and children study population stratified according to cfPWV percentiles.**

|  | **African-PREDICT study** | | | | **ExAMIN Youth SA study** | | | |
| --- | --- | --- | --- | --- | --- | --- | --- | --- |
|  | **Lower 15^th^ percentile**  (cfPWV≥ 5.5m/s)  (*n*= 197) | **Upper 85^th^ percentile**  (cfPWV≤ 7.2m/s)  (*n*= 175) | **Unadjusted**  ***p* value** | **Adjusted**  ***q* value** | **Lower 15^th^ percentile**  (cfPWV≥ 4.10m/s)  (*n*= 128) | **Upper 85^th^ percentile**  (cfPWV≤ 4.80m/s)  (*n*= 140) | **Unadjusted**  ***p* value** | **Adjusted**  ***q* value** |
| Ornithine (AU) | 7.47 (2.22; 22.4) | 7.92 (2.04; 17.7) | 0.682 | 0.70* | 596 (308; 1417) | 554 (278; 1210) | 0.229 | 0.99* |
| 5-Hydroxylysine (AU) | 10.2 (1.94; 31.2) | 10.8 (2.82; 25.5) | 0.533 | 0.63* | 424 (211; 997) | 455 (228; 1259) | 0.275 | 0.99* |
| Histidine (AU) | 745 (264; 2396) | 764 (231; 1829) | 0.701 | 0.70* | 18 374 (7259; 51978) | 17 567 (7808; 43317) | 0.650 | 0.99* |
| Lysine (AU) | 42.0 (14.8; 168) | 33.8 (12.2; 138) | **0.009** | **0.039*** | - | - | - | - |
| Asparagine (AU) | 26.7 (11.5; 90.4) | 23.4 (7.87; 54.1) | **0.038** | 0.099* | 19 761 (8039; 56337) | 19 800 (7614; 56279) | 0.978 | 0.99* |
| Arginine (AU) | 13.5 (5.92; 43.8) | 11.1 (5.98; 22.3) | **0.001** | **0.013*** | 15 870 (5562; 42218) | 14 457 (4654; 36440) | 0.182 | 0.99* |
| Glycine (AU) | 637 (169; 1249) | 490 (144; 1577) | **0.002** | **0.013*** | 6 777 (1873; 24829) | 6 461 (2020; 22983) | 0.630 | 0.99* |
| Dimethylglycine (AU) | 17.5 (2.39; 212) | 14.4 (2.87; 251) | 0.190 | 0.31* | 2 881 (650; 12205) | 2 697 (669; 16481) | 0.562 | 0.99* |
| Beta-alanine (AU) | 53.7 (4.56; 462) | 58.9 (4.23; 386) | 0.536 | 0.63* | 222 (98.5; 695) | 224 (113; 625) | 0.929 | 0.99* |
| Threonine (AU) | 96.0 (32.4; 369) | 77.1 (21.2; 195) | **0.005** | **0.024*** | - | - | - | - |
| Hydroxyproline (AU) | 3.53 (1.66; 9.52) | 3.77 (1.56; 14.4) | 0.632 | 0.70* | 411 (203; 1518) | 421 (190; 1466) | 0.784 | 0.99* |
| Citrulline (AU) | 5.16 (2.21; 23.2) | 4.64 (2.28; 12.5) | 0.176 | 0.31* | 164 (63.6; 696) | 146 (64.3; 519) | 0.207 | 0.99* |
| GABA(AU) | 0.28 (0.129; 1.21) | 0.24 (0.134; 0.494) | **0.019** | 0.057* | 101 (28.9; 4523) | 96.8 (31.1; 637) | 0.803 | 0.99* |
| Creatine (AU) | 24.0 (8.78; 134) | 16.2 (5.83; 70.8) | **<0.001** | **<0.001*** | 74 020 (7676; 427229) | 74 486 (64.3; 519) | 0.969 | 0.99* |
| Proline (AU) | 2.14 (1.00; 6.43) | 1.66 (0.679; 3.97) | **<0.001** | **<0.001*** | 380 (108; 983) | 360 (95.7; 1126) | 0.598 | 0.99* |
| Cystine (AU) | 0.71 (0.296; 2.91) | 0.64 (0.243; 0.175) | 0.185 | 0.31* | 147 (72.0; 339) | 145 (62.7; 309) | 0.803 | 0.99* |
| Valine (AU) | 5.80 (2.75; 17.9) | 4.80 (1.62; 10.5) | **0.005** | **0.024*** | 7 388 (3371; 17588) | 7 268 (2897; 15348) | 0.799 | 0.99* |
| Methionine (AU) | 1.30 (0.585; 3.97) | 1.13 (0.331; 2.75) | **0.035** | 0.098* | 591 (152; 1576) | 569 (152; 1323) | 0.632 | 0.99* |
| Tyrosine (AU) | 41.1 (18.0; 113) | 36.7 (11.3; 89.7) | 0.080 | 0.16* | 10 976 (4176; 22379) | 10 687 (3635; 21194) | 0.673 | 0.99* |
| Pyroglutamic acid (AU) | 27.2 (12.5; 80.7) | 24.5 (7.10; 59.6) | 0.086 | 0.16* | 5 528 (3230; 9586) | 5 468 (3393; 9502) | 0.855 | 0.99* |
| Leucine / isoleucine (AU) | 14.3 (6.00; 46.1) | 12.5 (3.83; 29.4) | 0.067 | 0.15* | 3 793 (1374; 8513) | 3 597 (1385; 7791) | 0.463 | 0.99* |
| Phenylalanine (AU) | 20.2 (7.83; 62.4) | 17.9 (5.67; 48.1) | 0.078 | 0.16* | 7 645 (2115; 16574) | 7 098 (2139; 18406) | 0.377 | 0.99* |
| Aspartic acid (AU) | 5.32 (2.77; 13.4) | 4.49 (1.67; 9.96) | **0.002** | **0.013*** | 2 516 (1110; 7006) | 2 557 (1028; 7384) | 0.836 | 0.99* |
| Tryptophan (AU) | 1.41 (0.428; 5.52) | 1.22 (0.34; 3.93) | 0.078 | 0.16* | 2 356 (859; 7417) | 2 246 (862; 6614) | 0.557 | 0.99* |
| Glutamic acid (AU) | 2.64 (1.12; 10.1) | 2.13 (0.705; 5.40) | **0.002** | **0.013*** | 22 209 (10172; 47455) | 22 561 (10001; 45714) | 0.845 | 0.99* |
| Serine (AU) | 265 (108; 764) | 225 (76.7; 553) | **0.013** | **0.026*** | 2 801 (1932; 12811) | 2 681 (1973; 12358) | 0.552 | 0.99* |
| Aminoadipic acid (AU) | 0.95 (0.277; 3.61) | 0.86 (0.239; 2.56) | 0.266 | 0.40* | 2 801 (1184; 8663) | 2 681 (968; 7473) | 0.552 | 0.99* |
| Free carnitine (AU) | 42.8 (15.8; 140.3) | 51.0 (17.9; 131) | **0.014** | **0.046*** | 11 471 (5421; 30156) | 11 779 (4983; 26446) | 0.660 | 0.99* |
| Acetylcarnitine (AU) | 10.4 (0.598; 95.6) | 12.1 (0.886; 78.2) | 0.316 | 0.44* | 1 463 (186; 12131) | 1 646 (150; 12099) | 0.516 | 0.99* |
| Propionylcarnitine (AU) | 1.43 (0.223; 9.46) | 1.97 (0.234; 10.3) | **0.012** | **0.046*** | 419 (38.9; 3506) | 447 (52.5; 2659) | 0.750 | 0.99* |
| Butyrylcarnitinen (AU) | 11.9 (3.28; 50.1) | 11.1 (2.83; 34.5) | 0.435 | 0.59* | 3 048 (1119; 8583) | 3 051 (820; 9199) | 0.993 | 0.99* |
| Isovalerylcarnitine (AU) | 0.54 (0.130; 2.62) | 0.59 (0.115; 2.29) | 0.275 | 0.40* | 242 (43.8; 1171) | 259 (49.0; 1196) | 0.618 | 0.99* |
| Hexanoylcarnitine (AU) | 0.07 (0.024; 0.281) | 0.06 (0.022; 0.221) | **0.044** | 0.11* | 59.5 (21.8; 643) | 55.4 (18.4; 645) | 0.629 | 0.99* |
| Octanoylcarnitine (AU) | 0.36 (0.155; 1.00) | 0.34 (0.167; 0.887) | 0.507 | 0.63* | 124 (47.7; 865) | 122 (46.1; 859) | 0.903 | 0.99* |
| Decanoylcarnitine (AU) | 0.22 (0.056; 0.863) | 0.21 (0.063; 0.605) | 0.564 | 0.65* | 84.8 (29.2; 1047) | 83.1 (25.2; 1035) | 0.887 | 0.99* |
| Dodecanoylcarnitine (AU) | 0.03 (0.012; 0.099) | 0.03 (0.011; 0.213) | 0.876 | 0.88* | 84.5 (36.5; 702) | 83.9 (36.6; 692) | 0.630 | 0.99* |

Values are arithmetic mean ± standard deviation or geometric mean (5th and 95th percentiles) for logarithmically transformed variables. * False discovery rate adjusted *p*-values. Abbreviations: *n* – number of participants. Bold values denote statistical significance (p<0.05).

**Supplementary Table S2. Pearson correlations of metabolites with markers of vascular stiffness in adult and children study population stratified according to cfPWV percentiles.**

|  | **African-PREDICT study** | | **ExAMIN Youth SA study** | |
| --- | --- | --- | --- | --- |
|  | **Lower 15^th^ percentile**  (cfPWV≥ 5.5m/s)  (*n*= 197) | **Upper 85^th^ percentile**  (cfPWV≤ 7.2m/s)  (*n*= 175) | **Lower 15^th^ percentile**  (cfPWV≥ 4.10m/s)  (*n*= 128) | **Upper 85^th^ percentile**  (cfPWV≤ 4.80m/s)  (*n*= 140) |
| Ornithine (AU) | r= –0.019;  *p*= 0.860 | r= 0.093;  *p*= 0.465 | r= –0.002;  *p*= 0.465 | r= –0.021;  *p*= 0.809 |
| 5-Hydroxylysine (AU) | r= –0.030;  *p*= 0.702 | r= –0.084;  *p*= 0.328 | r= –0.001;  *p*= 0.988 | r= –0.157;  *p*= 0.095 |
| Histidine (AU) | r= –0.058;  *p*= 0.414 | **r= –0.202;**  ***p*= 0.007** | r= –0.013;  *p*= 0.890 | r= 0.146;  *p*= 0.091 |
| Asparagine (AU) | r= –0.130;  *p*= 0.068 | r= –0.066;  *p*= 0.384 | r= –0.076;  *p*= 0.409 | r= 0.098;  *p*= 0.268 |
| Arginine (AU) | **r= –0.160;**  ***p*= 0.024** | r= –0.106;  *p*= 0.163 | r= –0.049;  *p*= 0.597 | r= 0.059;  *p*= 0.506 |
| Glycine (AU) | r= –0.063;  *p*= 0.380 | r= –0.104;  *p*= 0.169 | r= 0.032;  *p*= 0.726 | r= 0.084;  *p*= 0.334 |
| Dimethylglycine (AU) | r= –0.045;  *p*= 0.533 | r= 0.015;  *p*= 0.849 | r= 0.157;  *p*= 0.084 | r= 0.114;  *p*= 0.190 |
| Beta-alanine (AU) | r= –0.002;  *p*= 0.976 | **r= –0.171;**  ***p*= 0.024** | r= 0.060;  *p*= 0.513 | r= –0.041;  *p*= 0.634 |
| Hydroxyproline (AU) | r= –0.012;  *p*= 0.932 | r= –0.170;  *p*= 0.360 | r= –0.037;  *p*= 0.702 | r= –0.004;  *p*= 0.965 |
| Citrulline (AU) | r= –0.137;  *p*= 0.080 | r= –0.118;  *p*= 0.161 | r= –0.020;  *p*= 0.835 | r= –0.021;  *p*= 0.827 |
| GABA(AU) | r= –0.094;  *p*= 0.200 | r= –0.077;  *p*= 0.345 | r= 0.001;  *p*= 0.991 | r= –0.146;  *p*= 0.220 |
| Creatine (AU) | r= –0.100;  *p*= 0.162 | r= –0.115;  *p*= 0.129 | r= 0.039;  *p*= 0.674 | r= 0.043;  *p*= 0.623 |
| Proline (AU) | r= –0.007;  *p*= 0.927 | r= –0.047;  *p*= 0.538 | r= 0.024;  *p*= 0.792 | r= 0.050;  *p*= 0.564 |
| Cystine (AU) | r= –0.016;  *p*= 0.827 | r= –0.042;  *p*= 0.581 | r= 0.069;  *p*= 0.491 | r= 0.035;  *p*= 0.709 |
| Valine (AU) | r= –0.077;  *p*= 0.281 | r= –0.087;  *p*= 0.250 | r= 0.042;  *p*= 0.648 | r= 0.040;  *p*= 0.643 |
| Methionine (AU) | r= –0.128;  *p*= 0.075 | r= –0.079;  *p*= 0.303 | r= 0.067;  *p*= 0.465 | r= 0.047;  *p*= 0.592 |
| Tyrosine (AU) | r= –0.051;  *p*= 0.476 | r= –0.061;  *p*= 0.424 | r= –0.020;  *p*= 0.827 | r= 0.099;  *p*= 0.257 |
| Pyroglutamic acid (AU) | r= –0.078;  *p*= 0.277 | r= –0.062;  *p*= 0.413 | r= –0.054;  *p*= 0.554 | r= 0.150;  *p*= 0.083 |
| Leucine / isoleucine (AU) | r= –0.097;  *p*= 0.174 | r= –0.078;  *p*= 0.303 | r= 0.035;  *p*= 0.706 | r= 0.115;  *p*= 0.184 |
| Phenylalanine (AU) | r= –0.096;  *p*= 0.178 | r= –0.091;  *p*= 0.232 | r= 0.059;  *p*= 0.522 | **r= 0.172;**  ***p*= 0.046** |
| Aspartic acid (AU) | r= –0.078;  *p*= 0.278 | r= –0.075;  *p*= 0.322 | r= 0.055;  *p*= 0.548 | r= 0.025;  *p*= 0.774 |
| Tryptophan (AU) | r= –0.068;  *p*= 0.342 | r= –0.042;  *p*= 0.582 | r= –0.008;  *p*= 0.930 | r= 0.025;  *p*= 0.774 |
| Glutamic acid (AU) | r= –0.103;  *p*= 0.152 | r= –0.030;  *p*= 0.696 | r= –0.079;  *p*= 0.386 | r= 0.125;  *p*= 0.148 |
| Serine (AU) | r= –0.123;  *p*= 0.086 | r= –0.109;  *p*= 0.153 | r= 0.001;  *p*= 0.991 | r= 0.143;  *p*= 0.097 |
| Amioadipic acid (AU) | r= –0.032;  *p*= 0.651 | r= –0.019;  *p*= 0.801 | r= –0.074;  *p*= 0.461 | r= 0.022;  *p*= 0.798 |
| Free carnitine (AU) | r= –0.018;  *p*= 0.807 | r= –0.023;  *p*= 0.763 | r= –0.130;  *p*= 0.151 | r= 0.046;  *p*= 0.597 |
| Acetylcarnitine (AU) | r= 0.000;  *p*= 0.994 | r= –0.013;  *p*= 0.863 | r= –0.091;  *p*= 0.317 | r= 0.023;  *p*= 0.791 |
| Propionylcarnitine (AU) | r= –0.025;  *p*= 0.746 | r= –0.024;  *p*= 0.764 | r= –0.009;  *p*= 0.931 | r= –0.163;  *p*= 0.113 |
| Butyrylcarnitine (AU) | r= –0.028;  *p*= 0.694 | r= –0.100;  *p*= 0.188 | r= 0.002;  *p*= 0.981 | r= 0.042;  *p*= 0.630 |
| Isovalerylcarnitine (AU) | r= –0.087;  *p*= 0.224 | r= 0.009;  *p*= 0.904 | r= 0.000;  *p*= 0.998 | r= –0.016;  *p*= 0.858 |
| Hexanoylcarnitine (AU) | r= –0.110;  *p*= 0.127 | r= –0.049;  *p*= 0.518 | r= 0.033;  *p*= 0.730 | r= –0.100;  *p*= 0.270 |
| Octanoylcarnitine (AU) | r= –0.085;  *p*= 0.235 | r= –0.052;  *p*= 0.491 | r= 0.014;  *p*= 0.880 | r= –0.004;  *p*= 0.961 |
| Decanoylcarnitine (AU) | r= –0.093;  *p*= 0.192 | r= –0.086;  *p*= 0.258 | r= 0.043;  *p*= 0.644 | r= –0.066;  *p*= 0.458 |
| Dodecanoylcarnitine (AU) | r= –0.230;  *p*= 0.079 | r= –0.200;  *p*= 0.199 | r= 0.032;  *p*= 0.726 | r= –0.058;  *p*= 0.511 |

Abbreviations: *n* – number of participants. Bold values denote statistical significance (p<0.05).

**Supplementary Table S3. Partial correlations of metabolomics with markers of vascular stiffness in adult and children study population stratified according to cfPWV percentiles.**

|  | **African-PREDICT study** | | **ExAMIN Youth SA study** | |
| --- | --- | --- | --- | --- |
|  | **Lower 15^th^ percentile**  (cfPWV≥ 5.5m/s)  (*n*= 197) | **Upper 85^th^ percentile**  (cfPWV≤ 7.2m/s)  (*n*= 175) | **Lower 15^th^ percentile**  (cfPWV≥ 4.1m/s)  (*n*= 128) | **Upper 85^th^ percentile**  (cfPWV≤ 4.8m/s)  (*n*= 140) |
| Ornithine (AU) | r= –0.008;  *p*= 0.940 | r= 0.079;  *p*= 0.541 | r= 0.001;  *p*= 0.995 | r= –0.031;  *p*= 0.728 |
| 5-Hydroxylysine (AU) | r= –0.047;  *p*= 0.556 | r= –0.094;  *p*= 0.283 | r= 0.002;  *p*= 0.982 | r= –0.136;  *p*= 0.158 |
| Histidine (AU) | r= –0.040;  *p*= 0.579 | **r= –0.199;**  ***p*= 0.009** | r= –0.011;  *p*= 0.908 | r= 0.103;  *p*= 0.242 |
| Asparagine (AU) | r= –0.129;  *p*= 0.073 | r= –0.075;  *p*= 0.330 | r= –0.070;  *p*= 0.456 | r= 0.070;  *p*= 0.441 |
| Arginine (AU) | **r= –0.170;**  ***p*= 0.018** | r= –0.129;  *p*= 0.094 | r= –0.031;  *p*= 0.745 | r= 0.054;  *p*= 0.547 |
| Glycine (AU) | r= –0.095;  *p*= 0.107 | r= –0.095;  *p*= 0.216 | r= 0.010;  *p*= 0.916 | r= 0.043;  *p*= 0.624 |
| Dimethylglycine (AU) | r= –0.024;  *p*= 0.739 | r= 0.022;  *p*= 0.777 | r= 0.138;  *p*= 0.135 | r= 0.064;  *p*= 0.473 |
| Beta-alanine (AU) | r= –0.022;  *p*= 0.760 | **r= –0.183;**  ***p*= 0.016** | r= 0.068;  *p*= 0.464 | r= –0.032;  *p*= 0.720 |
| Hydroxyproline (AU) | r= –0.029;  *p*= 0.841 | r= –0.179;  *p*= 0.371 | r= –0.054;  *p*= 0.584 | r= –0.008;  *p*= 0.935 |
| Citrulline (AU) | r= –0.144;  *p*= 0.069 | r= –0.136;  *p*= 0.110 | r= –0.027;  *p*= 0.783 | r= –0.030;  *p*= 0.757 |
| GABA(AU) | r= –0.106;  *p*= 0.152 | r= –0.080;  *p*= 0.335 | r= 0.026;  *p*= 0.850 | r= –0.164;  *p*= 0.181 |
| Creatine (AU) | r= –0.088;  *p*= 0.226 | r= –0.129;  *p*= 0.093 | r= 0.049;  *p*= 0.596 | r= 0.025;  *p*= 0.776 |
| Proline (AU) | r= –0.030;  *p*= 0.683 | r= –0.048;  *p*= 0.532 | r= 0.022;  *p*= 0.811 | r= 0.028;  *p*= 0.756 |
| Cystine (AU) | r= –0.028;  *p*= 0.698 | r= –0.052;  *p*= 0.498 | r= 0.065;  *p*= 0.525 | r= 0.028;  *p*= 0.774 |
| Valine (AU) | r= –0.108;  *p*= 0.134 | r= –0.112;  *p*= 0.143 | r= 0.047;  *p*= 0.610 | r= 0.027;  *p*= 0.763 |
| Methionine (AU) | r= –0.124;  *p*= 0.088 | r= –0.069;  *p*= 0.372 | r= 0.060;  *p*= 0.521 | r= 0.032;  *p*= 0.717 |
| Tyrosine (AU) | r= –0.061;  *p*= 0.400 | r= –0.078;  *p*= 0.311 | r= –0.026;  *p*= 0.783 | r= 0.086;  *p*= 0.333 |
| Pyroglutamic acid (AU) | r= –0.086;  *p*= 0.233 | r= –0.060;  *p*= 0.433 | r= –0.073;  *p*= 0.434 | r= 0.109;  *p*= 0.217 |
| Leucine / isoleucine (AU) | r= –0.121;  *p*= 0.093 | r= –0.096;  *p*= 0.209 | r= 0.036;  *p*= 0.702 | r= 0.087;  *p*= 0.325 |
| Phenylalanine (AU) | r= –0.105;  *p*= 0.146 | r= –0.101;  *p*= 0.189 | r= 0.059;  *p*= 0.527 | r= 0.132;  *p*= 0.134 |
| Aspartic acid (AU) | r= –0.093;  *p*= 0.201 | r= –0.080;  *p*= 0.297 | r= 0.052;  *p*= 0.576 | r= 0.018;  *p*= 0.843 |
| Tryptophan (AU) | r= –0.089;  *p*= 0.217 | r= –0.069;  *p*= 0.069 | r= 0.007;  *p*= 0.936 | r= 0.009;  *p*= 0.918 |
| Glutamic acid (AU) | r= –0.116;  *p*= 0.109 | r= –0.033;  *p*= 0.664 | r= –0.073;  *p*= 0.430 | r= 0.092;  *p*= 0.297 |
| Serine (AU) | r= –0.109;  *p*= 0.131 | r= –0.108;  *p*= 0.159 | r= –0.008;  *p*= 0.933 | r= 0.105;  *p*= 0.234 |
| Amioadipic acid (AU) | r= –0.082;  *p*= 0.257 | r= –0.054;  *p*= 0.487 | r= –0.078;  *p*= 0.401 | r= –0.011;  *p*= 0.906 |
| Free carnitine (AU) | r= –0.071;  *p*= 0.326 | r= –0.056;  *p*= 0.467 | r= –0.116;  *p*= 0.210 | r= 0.059;  *p*= 0.507 |
| Acetylcarnitine (AU) | r= –0.053;  *p*= 0.462 | r= –0.054;  *p*= 0.483 | r= –0.080;  *p*= 0.388 | r= 0.032;  *p*= 0.716 |
| Propionylcarnitine (AU) | r= –0.054;  *p*= 0.484 | r= –0.047;  *p*= 0.559 | r= –0.012;  *p*= 0.914 | r= –0.134;  *p*= 0.204 |
| Butyrylcarnitine (AU) | r= –0.078;  *p*= 0.280 | r= –0.116;  *p*= 0.129 | r= 0.026;  *p*= 0.779 | r= 0.017;  *p*= 0.846 |
| Isovalerylcarnitine (AU) | **r= –0.143;**  ***p*= 0.048** | r= –0.037;  *p*= 0.634 | r= 0.000;  *p*= 0.997 | r= –0.006;  *p*= 0.947 |
| Hexanoylcarnitine (AU) | r= –0.131;  *p*= 0.072 | r= –0.059;  *p*= 0.449 | r= 0.027;  *p*= 0.784 | r= –0.082;  *p*= 0.373 |
| Octanoylcarnitine (AU) | r= –0.128;  *p*= 0.076 | r= –0.079;  *p*= 0.302 | r= 0.018;  *p*= 0.845 | r= –0.014;  *p*= 0.874 |
| Decanoylcarnitine (AU) | r= –0.125;  *p*= 0.084 | r= –0.114;  *p*= 0.137 | r= 0.033;  *p*= 0.724 | r= –0.058;  *p*= 0.525 |
| Dodecanoylcarnitine (AU) | r= –0.227;  *p*= 0.095 | r= –0.200;  *p*= 0.233 | r= 0.072;  *p*= 0.444 | r= –0.065;  *p*= 0.463 |

Adjusted for age, sex, ethnicity and mean arterial pressure. Abbreviations: *n* – number of participants. Bold values denote statistical significance (p<0.05).

**Supplementary Table S4:** **Standard multiple regression analyses with metabolomics and pulse wave velocity in the study population stratified according to cfPWV percentiles with adjustments made for priori covariates.**

|  | **Pulse wave velocity (m/s)** | | | | | |
| --- | --- | --- | --- | --- | --- | --- |
|  | **Lower 15^th^ cfPWV Percentile (*n*= 197)** | | | **Upper 85^th^ cfPWV Percentile (*n*= 175)** | | |
|  | **Adj R²** | **Std β (95 % Cl)** | ***p* value** | **Adj R²** | **Std β (95 % Cl)** | ***p* value** |
| Histidine (AU) | 0.006 | –0.039 (–0.065; 0.037) | 0.60 | 0.038 | –0.192 (–0.357; –0.043) | **0.013** |
| Beta-alanine (AU) | 0.005 | –0.015 (–0.057; 0.046) | 0.85 | 0.034 | –0.181 (–0.328; –0.030) | **0.019** |

Variables included in the models were: age, sex, ethnicity, BMI and mean arterial pressure. Abbreviations: *n* – number of participants. Bold values denote statistical significance (p<0.05).
